# Comparison of Cortical and White Matter Traumatic Brain Injury Models Reveals Differential Effects in the Subventricular Zone and Divergent Sonic Hedgehog Signaling Pathways in Neuroblasts and Oligodendrocyte Progenitors

**DOI:** 10.1177/1759091414551782

**Published:** 2014-09-17

**Authors:** Amanda J. Mierzwa, Genevieve M. Sullivan, Laurel A. Beer, Sohyun Ahn, Regina C. Armstrong

**Affiliations:** 1 Department of Anatomy, Physiology and Genetics, Uniformed Services University of the Health Sciences, Bethesda, MD, USA; 2 Center for Neuroscience and Regenerative Medicine, Uniformed Services University of the Health Sciences, Bethesda, MD, USA; 3 Program in Molecular and Cell Biology, Uniformed Services University of the Health Sciences, Bethesda, MD, USA; 4 Eunice Kennedy Shriver National Institute of Child Health and Human Development, National Institutes of Health, Bethesda, MD, USA

**Keywords:** neuroblast, oligodendrocyte progenitor, sonic hedgehog, stem cell, subventricular zone, traumatic brain injury

## Abstract

The regenerative capacity of the central nervous system must be optimized to promote repair following traumatic brain injury (TBI) and may differ with the site and form of damage. Sonic hedgehog (Shh) maintains neural stem cells and promotes oligodendrogenesis. We examined whether Shh signaling contributes to neuroblast (doublecortin) or oligodendrocyte progenitor (neural/glial antigen 2 [NG2]) responses in two distinct TBI models. Shh-responsive cells were heritably labeled *in vivo* using *Gli1-CreER^T2^;R26-YFP* bitransgenic mice with tamoxifen administration on Days 2 and 3 post-TBI. Injury to the cerebral cortex was produced with mild controlled cortical impact. Yellow fluorescent protein (YFP) cells decreased in cortical lesions. Total YFP cells increased in the subventricular zone (SVZ), indicating Shh pathway activation in SVZ cells, including doublecortin-labeled neuroblasts. The alternate TBI model produced traumatic axonal injury in the corpus callosum. YFP cells decreased within the SVZ and were rarely double labeled as NG2 progenitors. NG2 progenitors increased in the cortex, with a similar pattern in the corpus callosum. To further test the potential of NG2 progenitors to respond through Shh signaling, Smoothened agonist was microinjected into the corpus callosum to activate Shh signaling. YFP cells and NG2 progenitors increased in the SVZ but were not double labeled. This result indicates that either direct Smoothened activation in NG2 progenitors does not signal through *Gli1* or that Smoothened agonist acts indirectly to increase NG2 progenitors. Therefore, in all conditions, neuroblasts exhibited differential Shh pathway utilization compared with oligodendrocyte progenitors. Notably, cortical versus white matter damage from TBI produced opposite responses of Shh-activated cells within the SVZ.

## Introduction

Traumatic brain injury (TBI) is a heterogeneous injury most commonly resulting from direct impact to the head or forces from rapid acceleration–deceleration of the head. While many patients do recover substantially within a subacute period, it is clear that even without positive findings on a head computed tomography, many patients go on to experience chronic symptoms (Roozenbeek et al., 2013; McMahon et al., 2014). Even a single mild TBI will leave up to 33% of patients functionally impaired at 3 months postinjury, and many continue to have symptoms at 1 year (McMahon et al., 2014). The regenerative capacity of central nervous system (CNS) neural stem and progenitor cells must be optimized to promote repair and recovery following TBI, which can involve damage to multiple brain regions and cell types.

Endogenous neural stem cells of the subventricular zone (SVZ), a major germinal zone in the adult brain, have potential repair capacity that is not well understood relative to the heterogeneous forms of TBI. Promoting endogenous repair is particularly attractive in mild–moderate forms of TBI, for which stem cell therapy may not be required or appropriate. Sonic hedgehog (Shh) is a critical signaling pathway that maintains the neural stem cells in the adult SVZ (Palma et al., 2005; Balordi and Fishell, 2007; Petrova et al., 2013). Shh protein expression is maintained in the normal adult telencephalon and increases after cortical or white matter damage (Machold et al., 2003; Ferent et al., 2013; Sirko et al., 2013). Shh signaling is a complex pathway with many levels of modulation, which has been well detailed and illustrated in a recent review (Briscoe and Therond, 2013). In the canonical pathway, high levels of Shh binding to the Patched receptor lead to release of Smoothened (Smo) inhibition. Smo then travels to the primary cilium, where processing of GLI2 and GLI3 shifts to reduce repressor forms, mainly GLI3R, and accumulate activator forms, mainly GLI2A. GLI2/3 transcriptionally regulate *Gli1* that serves as an effective readout of high levels of Shh pathway activation. An important component of the regenerative response to injury in the adult CNS may then involve Shh signaling to maintain neural stem cell populations and stimulate the generation of neuroblasts or oligodendrocyte progenitors for the replacement of these respective cell lineages.

We used *in vivo* induction of genetic fate labeling to monitor the Shh-responsive cell population relative to neuroblasts and oligodendroglial progenitors following experimental TBI. Shh-responsive cells were heritably labeled in *Gli1-CreER^T2^* mice crossed to *R26-IAP* and *R26-YFP* reporter lines. Reporter expression is induced after tamoxifen administration, which enabled temporal control to fate label cells during the post-TBI period. The mosaic nature of the Cre recombination detects a relative ratio of expressing cells in a given population, rather than absolute numbers. In the normal adult mouse CNS, *Gli1-CreER^T2^* mice have provided important insights into the role of Shh in self-renewal and multipotentiality of neural stem cells and in regulating astrocytic phenotypes (Ahn and Joyner, 2005; Garcia et al., 2010; Ihrie et al., 2011). *Gli1-CreER^T2^* fate mapping of Shh pathway activation has not previously been studied in the context of CNS pathology.

We examined the SVZ repair potential after TBI and the contribution of the Shh signaling pathway based on induced genetic fate labeling in *Gli1-CreER^T2^* mice. Two different TBI models were used that produced either primarily gray matter or primarily white matter damage to determine whether the response to injury was specific to the site or cell type damaged. Controlled cortical impact (CCI) produced damage to the cerebral cortex. A mild severity of CCI was chosen to avoid cavitation and extension of the lesion into the corpus callosum. Traumatic axonal injury (TAI) produced a white matter injury with dispersed axonal injury throughout the corpus callosum (Sullivan et al., 2013). In both TBI models, the impact was centered at the coronal level of bregma to target regions near the SVZ. The data support a role for Shh signaling in both neuroblast and oligodendrocyte progenitor responses, with different downstream effectors of the pathway. Of particular note, the distinct injuries resulted in opposite responses of Shh-activated cells within the SVZ.

## Methods

### Heritable Labeling of Shh-Responsive Cells In Vivo

Mice were cared for according to the guidelines of the National Institutes of Health and the Institutional Animal Care and Use Committee of the Uniformed Services University of the Health Sciences. *Gli1-CreER^T2^* transgenic mice (*Gli1^tm3(cre/ESR1)Alj^/J*) contain a construct with tamoxifen-inducible Cre recombinase fused to the mutated estrogen receptor (CreER^T2^) that is expressed from the *Gli1* genomic locus in response to activation of the Shh pathway (Ahn and Joyner, 2004)*. Gli1-CreER^T2^* mice were crossed to either *R26-YFP* or *R26-IAP* mice, and first-generation heterozygotes were used for all experiments. The *R26-YFP* reporter mice (*B6.129X1-Gt(ROSA)26Sor^tm1(EYFP)Cos^/J*) contain the yellow fluorescent protein (YFP) reporter gene downstream of a floxed stop codon (Srinivas et al., 2001). The *R26-IAP* reporter mice (*B6;129-Gt(ROSA)26Sor^tm2Nat^/J*) constitutively express a floxed inverted second exon of the gene for the membrane-associated form of human alkaline phosphatase (AP), which accumulates in cell membranes after Cre-mediated recombination (Badea et al., 2009). To induce Cre-mediated expression of YFP or AP in Shh-responsive cells, 10 mg of tamoxifen (Sigma, St. Louis, MO) in corn oil was administered by oral gavage on Days 2 and 3 post-TBI (Sullivan et al., 2013).

### Controlled Cortical Impact

The CCI model was used to create a TBI model of gray matter damage created by direct impact onto the dura and cortex. Male (8–10 weeks old) *Gli1-CreER^T2^;R26-YFP* or *Gli1-CreER^T2^;R26-IAP* mice were anesthetized with isofluorane, and body temperature was maintained at 37℃. A craniotomy was performed to just exceed the size of the flat impact tip. The dura was impacted using an Impact One™ Stereotaxic Impactor device (Leica Biosystems; Buffalo Grove, IL) at 1.5 mm lateral (right hemisphere) to bregma using a tip diameter of 2 mm, a depth of 1 mm, a velocity of 1.5 m/s, and a dwell time of 100 ms. These parameters and the resulting cortical damage are classified as a mild form of the CCI model (Washington et al., 2012; Yi et al., 2012). The cortical cavitation does not extend down into the corpus callosum, but callosal cortical neurons are involved along with the corresponding axons in the corpus callosum. Sham animals underwent craniotomy without impact, and naïve animals did not receive anesthesia or surgery.

### Traumatic Axonal Injury

A previously characterized model of impact onto the closed skull was used to create white matter damage in the corpus callosum over the lateral ventricles (Sullivan et al., 2013). TAI was produced in male (8–10 weeks old) *Gli1-CreER^T2^;R26-YFP* or *Gli1-CreER^T2^;R26-IAP* mice, as previously detailed in C57BL/6 J mice (Sullivan et al., 2013). Briefly, impact to the skull was centered at bregma using an Impact One™ Stereotaxic Impactor device with a 3-mm diameter flat tip set to a depth of 1.5 mm, a velocity of 5 m/s, and a dwell time of 100 ms. Mice exhibited minimal linear skull fractures and no depressed skull fractures. This model results in TAI in the corpus callosum without hemorrhage and does not result in cortical cavitation (Sullivan et al., 2013). Sham mice received anesthesia as well as a scalp incision. Naive mice did not receive anesthesia or an incision.

### Smo Agonist Microinjection

*Gli1CreER^T2^;R26-YFP* mice were anesthetized with isofluorane and placed in a stereotaxic frame to drill a small hole in the skull for microinjection. A 10-µl Hamilton syringe (Cat# 7653-01; Hamilton Company, Reno, NV) with removable needle (RN) compression fitting adapters (Cat# 55750-01; Hamilton Company) to mount a pulled glass pipette (outer diameter 50 µm) was used to microinject 1 µl of Smo agonist (SAG; 10 µm; Cat# 566661; EMD Chemicals, San Diego, CA) or Hank's Balanced Salt Solution (HBSS) vehicle (GIBCO, Grant Island, NY) into the corpus callosum (1.25 mm to the right of bregma and 1.3 mm deep) for more than 5 min followed by 1 min of rest prior to the removal of the pipette. The skin was closed using Tissumend II SC (Veterinary Products Laboratories, Phoenix, AZ).

### Reporter Detection and Cell Type Identification

Mice were perfused with 3% paraformaldehyde at 3 days, 2 weeks, or 6 weeks after surgery, and brains were postfixed by immersion overnight. Coronal cryosections (14 µm) were processed either to detect AP activity or for immunohistochemistry. To detect AP activity, tissue sections from *Gli1-CreER^T2^;R26-IAP* mice were incubated in phosphate-buffered saline for heat inactivation of endogenous AP activity (69℃ for 90 min) and reacted with nitro-blue tetrazolium chloride (NBT)/5-bromo-4-chloro-3′-indolyphosphate p-toluidine salt (BCIP) substrate (DAKO, Carpinteria, CA) followed by methyl green nuclear counterstain (Vector Laboratories, Burlingame, CA). Tissue sections from *Gli1-CreER^T2^;R26-YFP* were immunostained with the following primary antibodies to detect β-amyloid precursor protein (βAPP; rabbit polyclonal, 1:100; Life Technologies, Grand Island, NY), doublecortin (DCX; rabbit polyclonal, 1:200; Cell Signaling, Danvers, MA), neural/glial antigen 2 (NG2; rabbit polyclonal, 1:500; gift from Dr. William Stallcup), Olig2 (rabbit polyclonal, 1:200; Millipore Billerica, MA), epidermal growth factor receptor (EGFR; sheep polyclonal, 1:5,000; Capralogics, Hardwick, MA), glial fibrillary acidic protein (GFAP; rabbit polyclonal, 1:500; DAKO), and S100β (mouse monoclonal, 1:1,000, clone SH-B1, Sigma). YFP reporter expression was detected with an antibody against green fluorescent protein (GFP, rat monoclonal, 1:100; Nacalai, Japan). Secondary antibodies used were as follows: donkey anti-rabbit IgG F(ab′)2 conjugated with Cy3 (Jackson ImmunoResearch, West Grove, PA) to detect βAPP; goat anti-rabbit IgG conjugated with Alexa Fluor 488 (Life Technologies, Grand Island, NY) to detect DCX, NG2, GFAP, and Olig2; donkey anti-mouse IgG F(ab′)2 conjugated with Cy3 (Jackson ImmunoResearch) to detect S100β; donkey anti-sheep IgG conjugated with Cy3 (Jackson ImmunoResearch) to detect EGFR; and goat anti-rat IgG conjugated with Alexa Fluor 555 (Life Technologies) to detect YFP. Sections were counterstained with 4′,6′-diamidino-2-phenylindole (DAPI; Sigma) before mounting with Vectashield (Vector Laboratories).

### Quantification and Statistical Analysis

Stereological quantification included three serial sections per mouse with four mice in each cohort. Each SVZ was outlined in the coronal sections (containing horizontal fibers of the anterior commissure; approximately +0.3 mm anterior to −0.2 mm posterior relative to bregma), and images were collected using the exhaustive grid setting in Stereo Investigator (MBF Bioscience, Williston, VT). The traced SVZ region was identified by DAPI nuclear staining to include cells along the edge of the lateral ventricle and extending from the dorsal SVZ less than 400 µm from the ventricle edge. Images were collected with the Z-stack interval of 1 µm. All YFP and DAPI-labeled cells were counted using a 50-µm × 50-µm grid, with a counting frame of 45 µm × 45 µm, placed over the outlined contours. All coefficient of error values (using Gunderson *m* = 1) were < .08.

AP-labeled cells and immunolabeled cells or axons were manually counted in regions of interest in at least three sections per mouse and three to four mice per condition in coronal brain sections containing horizontal fibers of the anterior commissure. For *Gli1-CreER^T2^;R26-YFP* mice, Spot Advanced (Sterling Heights, MI) was used to measure the area of the cerebral cortex (superior to the corpus callosum), corpus callosum, and SVZ, or the length of the SVZ extension. Images of coronal sections taken from *Gli1-CreER^T2^;R26-IAP* mice were collected using a NanoZoomer 2.0-RS (Hamamatsu, Hamamatsu, Japan), and the area of the cortex was measured using Metamorph (Molecular Devices, Downington, PA).

Prism 6.0 (GraphPad Software) was used for graphing and statistical analysis. βAPP in the corpus callosum was analyzed using one-way analysis of variance followed by a Dunnett’s post hoc analysis for multiple comparisons. Two-way analysis of variance was performed to determine significant differences between injured and sham or naïve cohorts across multiple conditions, such as time point and side (for injury or injection), followed by Tukey’s post hoc analysis for significant interactions. Statistical significance was determined as *p* < .05. Unless otherwise stated, interactions and effect sizes with a *p* > .05 are not specified.

## Results

### Heritable Labeling of Shh-Responsive Cells After Mild CCI

**Figure 1.**
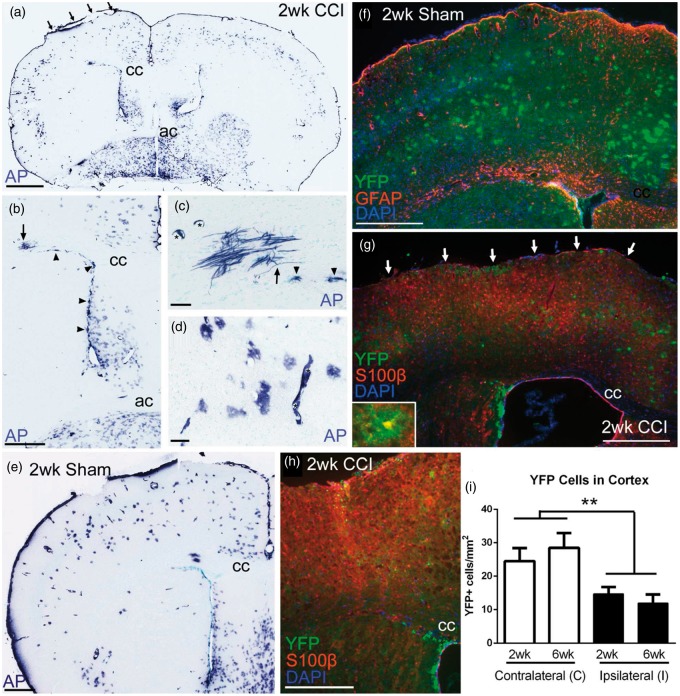
Shh-responsive cells heritably labeled after cortical injury from mild CCI. (a–e) *Gli1-CreER^T2^;R26-IAP* mice show strong AP labeling of multiple cell types in coronal brain sections. Mice were administered tamoxifen on Days 2 and 3 followed by survival until 2 weeks post-CCI (a, arrows indicate site of impact on dura) or postsham surgery (e). This heritable labeling identifies cells that were responding to high levels of Shh to drive *Gli1* transcription during tamoxifen-induced nuclear translocation of the Cre-ER fusion protein. AP-labeled cells are abundant in the SVZ (arrowheads in b, c). Rare AP-labeled myelinating oligodendrocytes have processes that make characteristic T-intersections to extend along axons (arrows in b, c). AP-labeled cells with the morphology of astrocytes are common in the cerebral cortex (d). AP labeling is also observed in Shh-responsive endothelial cells of blood vessels (asterisks in c, d). Examples in (c) and (d) are from sections adjacent to (a) and (b). (f–h) *Gli1-CreER^T2^;R26-YFP* mice show a similar pattern of heritable YFP labeling as with AP at 2 weeks postsurgery in sham (e, f) and CCI mice (a, g). GFAP immunolabeling demonstrates an astrocytic reaction to craniotomy in sham mice (g). GFAP does not consistently label the population of cortical astrocytes that express YFP (g). Cortical astrocytes with thin, highly branched processes are double labeled with YFP and S100β (g, inset). Not all S100β cells are labeled with YFP, particularly reactive astrocytes in the lesion and penumbra (g, arrows indicate site of impact on dura). YFP cells that remain in the lesion and penumbra maintain a morphology with fine, complex processes that is distinct from the reactive astrocytes labeled for S100β but not YFP (h). Nuclear DAPI colocalization was used to differentiate intact YFP cells from debris or autofluorescence in lesions (h). (i) At both 2 and 6 weeks post-CCI, YFP-labeled cells are reduced in the cortex under the site of impact (injury effect, **p = .0023, *n* = 4 mice per condition). cc = corpus callosum, ac = anterior commissure. Scale bars: a = 1 mm; b, e = 500 µm; c, d = 50 µm; f to h = 250 µm.

We performed a mild CCI injury at the coronal level of bregma to facilitate simultaneous evaluation of the cortical lesion area along with the SVZ (Figure 1). Shh-responsive cells were fate labeled using *Gli1-CreER^T2^* driver line of mice crossed to two different reporter lines. Prior studies of cortical cryoinjury or stab wound demonstrated that Shh signaling and protein levels were increased at 3 days postinjury (Amankulor et al., 2009; Sirko et al., 2013). Thus, we administered tamoxifen on Days 2 and 3 postinjury to induce reporter expression during the acute Shh response and then evaluated those heritably labeled cells at 2 and 6 weeks postinjury. *R26-IAP* reporter mice were used to generate strong labeling that could be evaluated in the overall tissue sections based on cell location and morphology. At 2 weeks after CCI (Figure 1(a)) or sham surgery (Figure 1(e)), *Gli1-CreER^T2^;R26-IAP* mice exhibited AP reaction indicative of Shh pathway activation through *Gli1* transcription in cells of the SVZ (Figure 1(b)) and cortical astrocytes (Figure 1(d)) with rare labeling of oligodendrocytes (Figure 1(c)).

### Cortical Astrocyte Response to Mild CCI

We then crossed the *Gli1-CreER^T2^* mice with *R26-YFP* reporter mice to enable immunohistochemical double labeling of Shh-responsive cells with specific cell type markers. The astrocytic response to injury was examined with GFAP and S100β (Figure 1(f) and (g)). YFP cells with the finely branched morphology of cortical astrocytes were more consistently double labeled with S100β (Figure 1(g), inset), confirming the findings of Garcia et al. (2010) that S100β is a more useful marker of the *Gli1*-labeled astrocyte population than GFAP. Increased GFAP was evident in sham mice, indicating reaction to the craniotomy (Figure 1(f)). The CCI mice exhibited dense reactive astrocytes in the lesion and penumbra that express GFAP (data not shown) and S100β but do not express YFP (Figure 1(g) and (h)). In fact, in both reporter lines, the density of AP- or YFP-labeled cells was dramatically reduced in cortical lesions (Figure 1(a), (g), and (h)). In the *Gli1-CreER^T2^;R26-YFP* mice, YFP-labeled cells were significantly reduced in the cerebral cortex ipsilateral to impact at both 2 and 6 weeks postinjury (Figure 1(i)). Furthermore, *Gli1* fate-labeled cells that were present in the lesion and penumbra maintained a finely branched morphology and did not transition to a reactive morphology. This finding illustrates that the YFP-labeled population of cortical astrocytes, although often labeled by S100β, exhibits a distinctly different response to injury compared with the S100β expressing population of reactive astrocytes that fill the lesion area.

### Shh-Responsive Cells in the SVZ After Mild CCI

**Figure 2.**
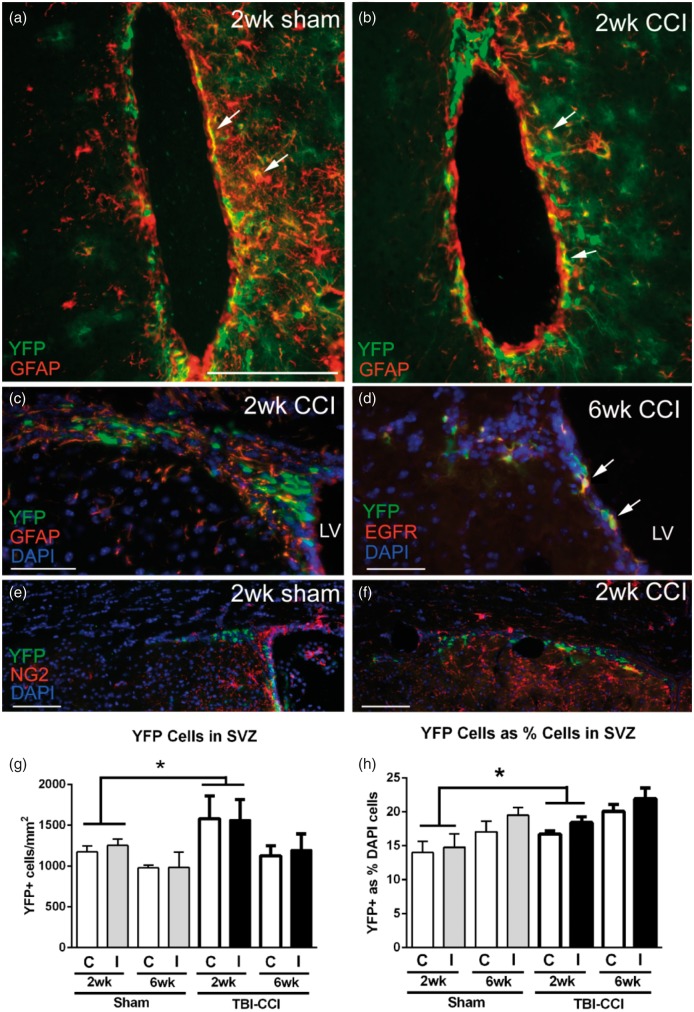
Shh-responsive cells are increased in the SVZ after CCI. Immunohistochemistry on coronal sections in *Gli1-CreER^T2^;R26-YFP* mice to examine *Gli1* fate-labeled cells relative to markers for distinct SVZ cell populations after mild CCI. (a, b) At 2 weeks post-CCI in the ventral SVZ of both sham (a) and CCI (b) mice, YFP cells can be found double labeled with GFAP (arrows), a marker of SVZ neural stem cells. (c) However, in the dorsolateral SVZ, YFP cells were not typically double labeled for GFAP at 2 weeks post-CCI. (d) YFP cells often express EGFR, indicating fate labeling of transit amplifying cells through at least 6 weeks post-CCI. Cells double labeled with YFP (arrows) shown in the SVZ just below the dorsolateral angle. (e, f) NG2-immunolabeled oligodendrocyte progenitors appear to be a distinct population from the YFP-labeled cells in the SVZ of sham (e) or CCI (f) mice. (g) The density of YFP cells is increased in the SVZ at 2 weeks (injury effect,*p = .0339) but not at 6 weeks (injury effect, *p* = .3417). (h) Further quantification using stereological methods to compare YFP cells as a percentage of total cells (DAPI nuclear marker) in the SVZ also shows an increase at 2 weeks (injury effect, *p = .0388) but not at 6 weeks (injury effect, *p* = .0648). The percent YFP cells on the ipsilateral side (I) is significantly increased compared with the contralateral side (C) in the CCI mice (side effect, *p* = .0051) but not the sham mice (side effect, *p* = .2339). LV = lateral ventricle. Scale bars: a and b = 100 µm; c and d = 50 µm; e and f = 100 µm; g, h; *n* = 4 mice per condition.

*Gli1* is actively transcribed in slow cycling neural stem cells in the noninjured adult SVZ (Petrova et al., 2013). To examine the response to injury, genetic fate labeling of Shh-responsive cells in the SVZ was examined at 2 and 6 weeks post-CCI and in matched sham *Gli1-CreER^T2^;R26-YFP* mice (Figure 2). Cells located mainly in the ventral SVZ labeled for both YFP and GFAP, a marker of quiescent (type B) neural stem cells, at the 2-week time point (Figure 2(a) to (c)). Double labeling for YFP and EGFR indicated *Gli1* fate labeling of transit amplifying (type C) cells at 6 weeks post-CCI (Figure 2(d)). This pattern appeared to be consistent in both CCI and sham mice. Therefore, tamoxifen administration on Days 2 and 3 post-CCI resulted in *Gli1* fate labeling of neural stem cells that gave rise to transit amplifying cells through at least 6 weeks post-CCI. The YFP population in the SVZ did not appear to colocalize with oligodendrocyte NG2 progenitor cells (Figure 2(e) and (f)). YFP cells in the SVZ were significantly increased at 2 weeks after CCI (Figure 2(g)). Additional analysis using stereology to quantify the YFP cells relative to the overall SVZ population (DAPI + cells) also showed that the proportion of cells labeled with YFP was significantly increased at 2 weeks after CCI (Figure 2(h)). The YFP proportion was increased significantly on the ipsilateral side in the CCI mice but not the sham mice (Figure 2(h)). In addition, the YFP cell proportion on the contralateral side of the sham mice was similar to values in age-matched naïve mice (2-week sham = 14.0 ± 1.6, naïve = 15.4 ± 1.1; 6-week sham = 17.0 ± 1.5, naïve = 15.6 ± 1.1; *n* = 4 mice per condition; *p* = .9939). Therefore, cortical injury stimulated an increase of Shh-responsive cells in the SVZ.

### Neuroblast Response to Mild CCI

**Figure 3.**
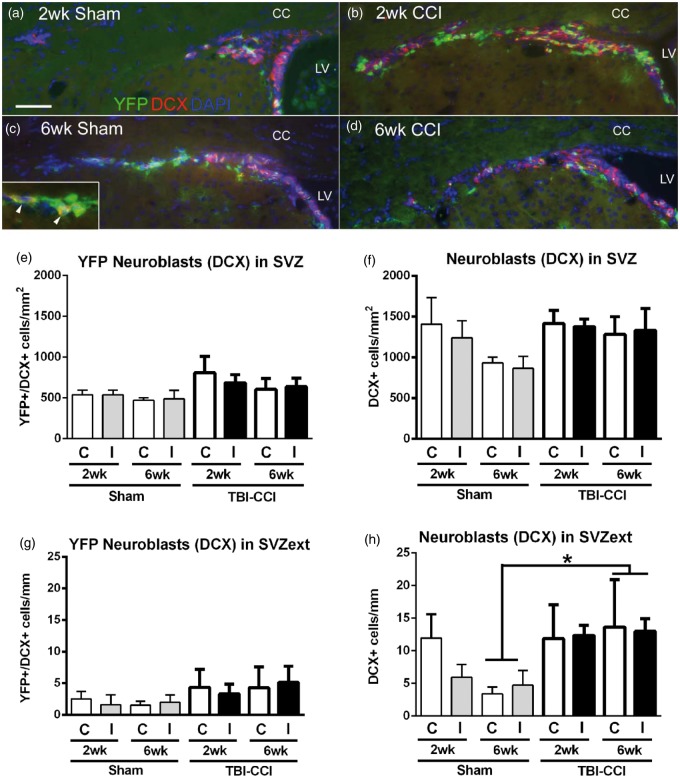
Shh pathway activation in neuroblasts after CCI. Immunohistochemistry of coronal sections from *Gli1-CreER^T2^;R26-YFP* mice to examine *Gli1* fate-labeled cells relative to the DCX marker for neuroblasts after CCI. (a–d) Cells within the ipsilateral SVZ and extending out from the dorsolateral SVZ are labeled with YFP (green), DCX (red), and DAPI (blue). YFP-labeled cells are often double labeled for DCX (c, inset arrowheads). (e–h) Quantification of YFP and DCX immunolabeling in the SVZ (e), (f) and in the extension from the dorsolateral SVZ (g, h, SVZext, i.e., > 400 µm from the lateral ventricle edge). Analysis of the subset of YFP cells double labeled for DCX had an overall increase following CCI that was highest at 2 weeks, although not significantly different in the SVZ (e, injury effect, *p* = .1114) or SVZ extension (g, injury effect, *p* = .3631). The total number of DCX neuroblasts showed the greatest difference between injured and sham at 6 weeks in both the SVZ (f, injury effect, *p* = .0529) and SVZ extension (h, injury effect,*p = .0376). Among sham mice, the reduction of DCX cells in the SVZ is not significantly different between time points (*p* = .0665). CC = corpus callosum, C = contralateral to CCI or sham craniotomy, I = ipsilateral, LV = lateral ventricle. Scale bar: 100 µm. e to h; *n* = 4 mice per condition.

We next examined whether cortical injury stimulated a potential regenerative response in neuroblasts. In normal adult *Gli1-CreER^T2^;R26-YFP* mice, the majority of YFP cells generated from the SVZ was neuroblasts identified by DCX (Ihrie et al., 2011). After both CCI and sham surgery, DCX neuroblasts were associated with the SVZ (Figure 3(a) to (d)) and were not observed in the cerebral cortex, including the lesion and penumbra (data not shown). DCX cells within the SVZ exhibited double labeling with YFP, but the density of double-labeled cells was not significantly increased after CCI (Figure 3(e)). Analysis of total DCX cells, regardless of YFP status, in the SVZ also did not show a significant difference (Figure 3(f)). After CCI, cells appeared to more frequently be found extending (>400 µm) from the dorsolateral SVZ. In this extension, overall DCX cells were significantly increased at 6 weeks after CCI, although the subset of DCX cells double labeled with YFP was not significantly different (Figure 3(g) and (h)). Thus, Shh-responsive cells within the SVZ included neuroblasts. However, the neuroblast response to mild CCI was less robust than the response of the overall population of Shh-responsive cells.

### Heritable Labeling of Shh-Responsive Cells After Corpus Callosum TAI

**Figure 4.**
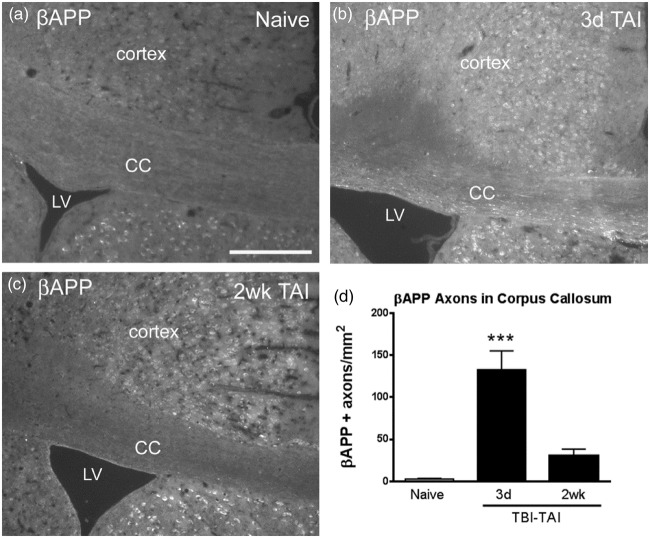
Closed skull impact produces traumatic axonal injury (TAI) in the corpus callosum. (a–c) Coronal sections through the corpus callosum of *Gli1-CreER^T2^;R26-YFP* mice immunostained for βAPP to detect impaired axonal transport, indicating TAI. βAPP is present in the cytoplasm of cortical neuron cell bodies (a to c) and is markedly increased in axons of the corpus callosum at 3 days post-TBI (b). (d) βAPP axonal profiles are significantly increased at 3 days post-TAI with return to near baseline by 2 weeks post-TAI (***p = .0002 across time points, with *p* < .05 for post-hoc comparison of 3-day TAI to naïve; *n* = 3 mice per condition). Scale bar: a = 200 µm. CC = corpus callosum, LV = lateral ventricle.

To determine whether the SVZ response differs between cortical damage and white matter injury, we next examined the *Gli1-CreER^T2^* mice using a model of axon damage in the corpus callosum adjacent to the SVZ. This TAI model does not stimulate proliferation within the overall population of SVZ cells but does induce a proliferative response in NG2 oligodendrocyte progenitors in the SVZ and corpus callosum at 3 days postinjury (Sullivan et al., 2013). Because prior characterization of this TAI model was in C57BL/6 mice, we first confirmed that the closed skull impact generated appropriate TAI within the corpus callosum of *Gli1-CreER^T2^* mice, which are on an outbred genetic background. βAPP immunohistochemistry detected impaired axonal transport in the corpus callosum of *Gli1-CreER^T2^;R26-YFP* mice (Figure 4). βAPP accumulations were significantly increased at 3 days after TAI (Figure 4(d)). Therefore, we administered tamoxifen on Days 2 and 3 postinjury to capture the Shh response during ongoing axonal damage and to facilitate comparison of the same time points as used in the mild CCI study.

**Figure 5.**
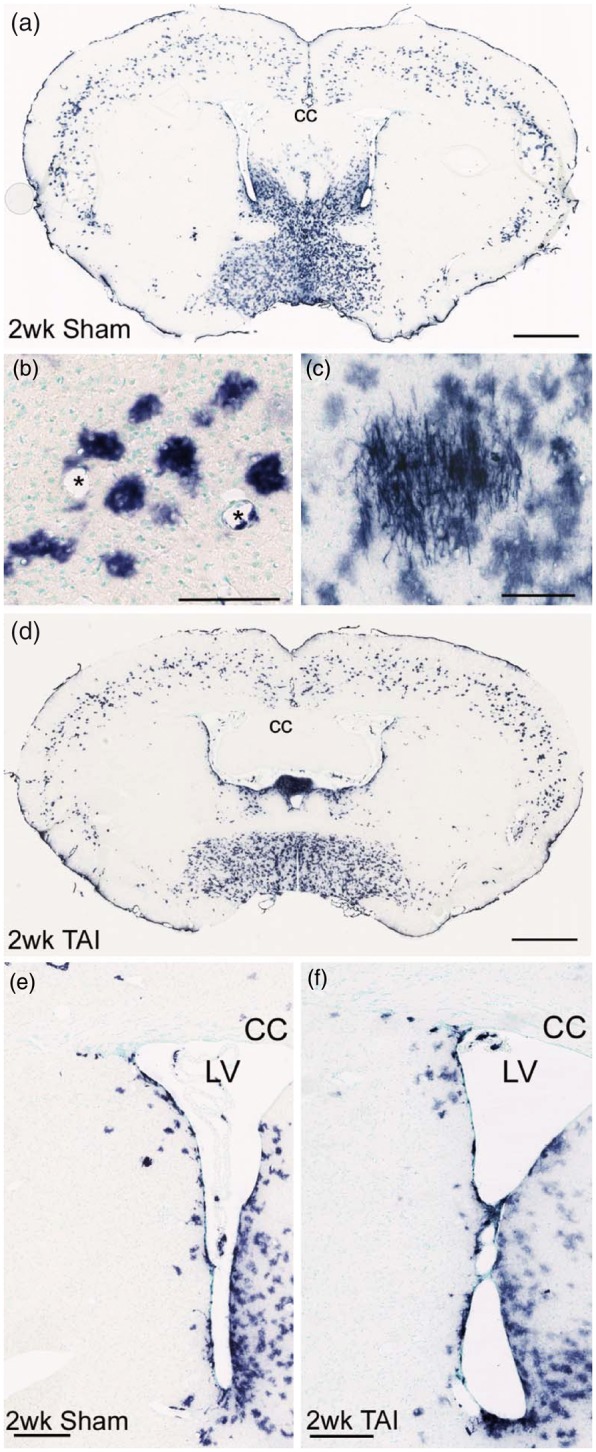
Shh-responsive cells heritably labeled with the AP reporter after TAI from closed head impact. AP reaction in coronal sections from *Gli1-CreER^T2^;R26-IAP* mice administered tamoxifen on Days 2 and 3 after surgery and analyzed at 2 weeks post-TAI or sham surgery. (a) Sham mouse showing AP labeling of cells in the SVZ, cerebral cortex, and ventral forebrain. (b) AP labeling in sham mouse illustrating AP labeling in cortical cells with an astrocytic morphology. Blood vessels marked by asterisks. (c) *Gli1* heritably labeled cells were rarely present in white matter, although labeling of cells with the morphology of myelinating oligodendrocytes was easily detected by AP processes aligned parallel to axons. Examples in (b) and (c) are from adjacent sections. (d) TAI mouse showing a similar pattern of AP labeling, as compared with sham (a). (e, f) Shh-responsive cells were abundant in the SVZ of sham (e) and TAI mice (f). CC = corpus callosum, LV = lateral ventricle. Scale bars: a = 1 mm; b = 100 µm; c = 100 µm; d = 1 mm; e = 200 µm; f = 250 µm.

The *Gli1-CreER^T2^;R26-IAP* mice were used for an overall evaluation of Shh-responsive cells after TAI in the corpus callosum. AP labeling in sham mice was similar to that observed in TAI mice (Figure 5). In contrast to the CCI model, after TAI, the density of cortical AP-labeled cells (Figure 5(b)) was not significantly different from sham (Figure 5(a) and (d); 2-week sham = 55.34 ± 6.03; 2-week TAI = 54.80 ± 6.17; *p* = .9526). Based on the localization of damage to the white matter, it was also of particular interest to examine the oligodendrocyte lineage and myelin repair. The membrane-bound AP reporter accumulates in myelin sheaths and facilitates the detection of oligodendrocytes, which can be seen in the example of an oligodendrocyte cluster observed in the basal forebrain (Figure 5(c)). However, AP-labeled cells with an oligodendrocyte morphology were rarely detected, even in areas of TAI in the corpus callosum (Figure 5(d)). In the SVZ, the overall distribution of *Gli1* fate-labeled cells was not markedly different after TAI (Figure 5(e) and (f)).

### Shh-Responsive Cells in the SVZ After Corpus Callosum TAI

**Figure 6.**
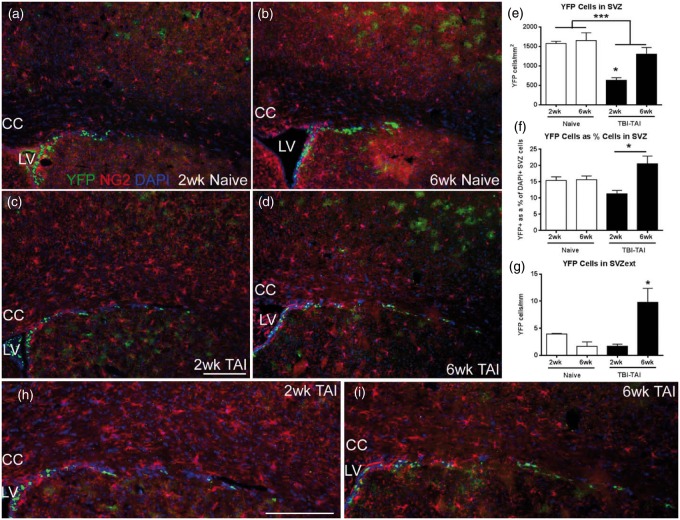
TAI in the corpus callosum alters SVZ cells. (a–d) Closed head impact TBI *Gli1-CreER^T2^;R26-YFP* mice, and age-matched naïves, were administered tamoxifen on Days 2 and 3 after injury followed by analysis at 2 and 6 weeks after injury. Coronal sections immunolabeled for NG2 (red) to identify oligodendrocyte progenitors and YFP (green) as the reporter of the heritable *Gli1* labeling, with DAPI nuclear stain (blue). (e) YFP cell density is decreased in the SVZ at 2 weeks after TAI compared with naïve and is normalized by 6 weeks, resulting in a significant effect of both time and injury (e, injury effect, ***p = .0009; time effect, *p* = .0229, *p < .01 for 2 weeks vs. 6 weeks post-TAI; for each time point, *n* = 3 naïve, *n* = 4 TAI). (f) Stereological quantification of YFP-labeled cells relative to the density of DAPI nuclei in the SVZ showed a similar pattern (*p < .01 for 2 weeks vs. 6 weeks post-TAI; interaction, *p* = .0119, time effect, *p* = .0087; *n* = 4 mice per condition). (g) to (i) In the dorsolateral SVZ extension (h, i, enlarged views from c, d), YFP cells were significantly increased at 6 weeks post-TAI (interaction effect, *p* = .0081; *p < .05 for 6 weeks post-TAI compared with either 2 weeks post-TAI or 6 week naïve; for each time point, *n* = 3 naïve, *n* = 4 injured). CC = corpus callosum, LV = lateral ventricle. Scale bars: c, h = 200 µm.

For more specific analysis of the SVZ response to TAI, the YFP-labeled population in *Gli1-CreER^T2^;R26-YFP* mice was examined in combination with immunostaining for NG2 oligodendrocyte progenitors (Figure 6(a) to (d)). Surprisingly, YFP double labeling with NG2 was extremely rare in the SVZ, representing less than 2% of the YFP cells in naïve mice and was further reduced after TAI (2-week TAI = 2.4 ± 1.5, naïve = 17.0 ± 3.9 cells/mm^2^; 6-week TAI = 12.7 ± 6.1, naïve = 34.6 ± 17.5 cells/mm^2^; injury effect, *p* = .0544). Therefore, quantitative analysis of the heritably labeled cells is shown for the total YFP population (Figure 6(e) to (g)). In this way, the total YFP population analysis after TAI (Figure 6(e) and (f)) is matched to the analysis after CCI (Figure 2(g) and (h)). The CCI analysis was compared with sham surgery for the important consideration of a craniotomy effect in the CCI model. TAI mice do not receive craniotomy, and so comparison with naïve mice provides a baseline of YFP labeling that can be used as a common point for analysis of either the CCI or the TAI mice.

The YFP-labeled cell density was significantly decreased in the SVZ at 2 weeks postinjury but not at 6 weeks (Figure 6(e)). A similar pattern was observed with stereological quantification of YFP-labeled cells as a proportion of the overall cell population within the SVZ (Figure 6(f)). A more marked increase of YFP cells occurred at 6 weeks in the region of the SVZ dorsolateral extension (Figure 6(g) to (i)).

### Oligodendrocyte Progenitor Response to Corpus Callosum TAI

**Figure 7.**
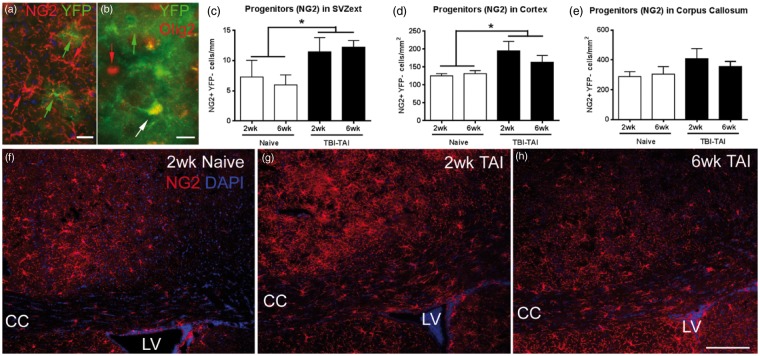
TAI in the corpus callosum alter oligodendrocyte progenitors. Closed head injury in *Gli1-CreER^T2^;R26-YFP* mice, and age-matched naïves, administered tamoxifen on Days 2 and 3 after injury, followed by analysis at 2 and 6 weeks after injury. (a) Coronal sections immunolabeled for NG2 (red) to identify oligodendrocyte progenitors and YFP (green) as the reporter of the heritable *Gli1* labeling, with DAPI nuclear stain (blue). NG2 progenitors were only rarely heritably labeled with YFP, as shown in this enlarged view from the cortex, 6 week-TAI mouse. (b) Olig2, a broad marker of the oligodendrocyte lineage, also only rarely double-labeled YFP cells, 6-week TAI mouse. (c to e) NG2 progenitors extending from the dorsolateral SVZ (SVZext) were increased at 2 and 6 weeks after TAI (c, injury effect, *p = .0283). After TAI, NG2 progenitors were also significantly increased in the cortex (d; injury effect, *p = .0307), with a similar pattern in the corpus callosum that did not reach significance (e; injury effect, *p* = .1243). *n* = 3 naïve, *n* = 4 injured. (f) to (h) NG2 cells in the cortex and corpus callosum in naïve (f), TAI 2 weeks (g), and TAI 6 weeks. CC = corpus callosum, LV = lateral ventricle. Scale bars: a = 20 µm; b = 10 µm; h = 200 µm.

Oligodendrocyte progenitors, immunolabeled with NG2, were rarely heritably labeled in *Gli1-CreER^T2^;R26-YFP* mice, even in the corpus callosum or cortical regions that could be affected after TAI (Figures 6(a) to (d), (h) and (i), and 7(a)). Immunolabeling with Olig2 as a broad marker of the oligodendrocyte lineage also rarely identified cells double labeled with YFP (Figure 7(b)). However, oligodendrocyte lineage cells that were infrequently labeled for *Gli1* transcriptional activation could, in fact, differentiate into mature myelinating oligodendrocytes, as was evident by AP labeling of myelin sheaths in *Gli1-CreER^T2^;R26-IAP* mice (Figure 5(c)).

TAI in the corpus callosum increased NG2 progenitors in multiple regions (Figure 7(c) to (h)). After TAI, NG2 progenitors were significantly increased in the SVZ extension (Figure 7(c)) and in the cortex (Figure 7(d)). NG2 progenitors in the corpus callosum were also increased, but this difference was not significant (Figure 7(e)).

**Figure 8.**
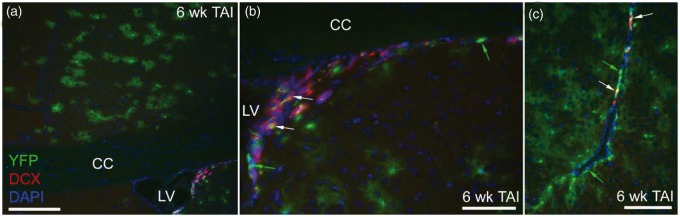
Gli1 fate-labeled neuroblasts persist in the SVZ after TAI. Immunohistochemistry on coronal sections in *Gli1-CreER^T2^;R26-YFP* mice to examine *Gli1* fate-labeled cells relative to the DCX marker for neuroblasts at 6 weeks after TAI. (a) DCX expressing cells are present in the SVZ but are not observed in the overlying corpus callosum. (b, c) The dorsal (b) and ventral (c) SVZ contain cells labeled for YFP without DCX (green arrows) and with DCX double labeling (white arrows). CC = corpus callosum, LV = lateral ventricle. Scale bars: a = 200 µm; b and c = 100 µm.

TAI did not result in DCX neuroblasts in the corpus callosum (Figure 8(a)). DCX neuroblasts are present in the dorsal and ventral SVZ out to 6 weeks post-TAI (Figure 8(b) and (c)). These findings illustrate the difference between the DCX neuroblasts and NG2 progenitors and provide further evidence of distinct differences in *Gli1* transcriptional activation between the neuronal and oligodendroglial lineages.

### Response of SVZ and Corpus Callosum Cells to SAG Microinjection

Pharmacological activation of the Shh pathway with SAG microinjection was used for comparison with the opposing effects observed among YFP cells in the SVZ after CCI versus TAI. In addition, the almost complete absence of YFP cells in the corpus callosum was striking, given that CCI causes cortical damage to callosal projection neurons, and TAI damages axons within the corpus callosum itself. Therefore, this pharmacological activation was targeted to the corpus callosum to test for *Gli1* transcriptional activation and heritable labeling.

**Figure 9.**
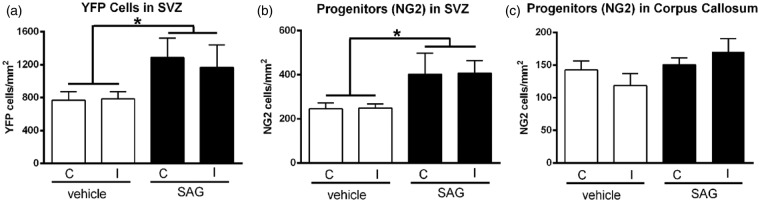
Pharmacological activation of Shh signaling. Naïve *Gli1-CreER^T2^;R26-YFP* mice were administered SAG, an Smo agonist, to examine the effect of pharmacological activation of the Shh pathway. SAG was microinjected into the corpus callosum. Tamoxifen was administered on Days 2 and 3 after SAG or vehicle injection. Mice were perfused for tissue analysis at 2 weeks after injection. (a) SAG increased the number of YFP cells in the SVZ on both the ipsilateral (I) and contralateral (C) sides (treatment effect, *p = .0376). (b) NG2 cells in the SVZ are also increased with SAG administration (treatment effect, *p = .0181). (c) Within the corpus callosum, the increase of NG2 after SAG administration is not significantly different from the vehicle condition (treatment effect, *p* = .0893). *n* = 4 mice per condition. SAG did not induce detectable YFP expression in NG2 cells (data not shown).

We injected SAG into the corpus callosum over the ventricle in noninjured adult *Gli1-CreER^T2^;R26-YFP* mice. Tamoxifen was administered on Days 2 and 3 with tissue analysis at 2 weeks postinjection. SAG injection significantly increased YFP labeling in cells of the SVZ, demonstrating effective activation of the Shh pathway through Smo and GLI1 (Figure 9(a)). Similarly, after SAG injection, a significant increase of NG2 progenitors was observed in the SVZ (Figure 9(b)) along with an increase of NG2 cells within the corpus callosum that was not a significant difference (Figure 9(c)). NG2 cell density in the cortex was similar after the injection of SAG or vehicle (SAG ipsi = 64.91 ± 11.96, contra = 64.22 ± 11.45; vehicle ipsi = 67.71 ± 15.00, contra = 66.70 ± 11.31 cells/mm^2^; treatment effect, *p* = .6807; injection side effect, *p* = .8942). Therefore, even in the SVZ where NG2 cells were increased after SAG injection, NG2 cells did not exhibit a corresponding increase of *Gli1* transcriptional activation. Furthermore, SAG injection did not result in YFP labeling of either NG2 progenitors or other cell types within the corpus callosum.

## Discussion

CNS damage mobilizes diverse populations of neural stem and progenitor cells. Immature neural cells that persist in the adult CNS often increase proliferation and take on developmental characteristics in lesion environments. The induced genetic fate mapping approach used in the current study enabled YFP or AP heritable labeling of cells transcribing *Gli1* as an indicator of Shh pathway activation in the early post-TBI period. To our knowledge, this is the first study to monitor the *in vivo* activation of a specific signaling pathway in the SVZ after TBI. This approach using *Gli1-CreER^T2^* driver mice reveals opposite effects among Shh-responsive cells in the SVZ following CNS damage in the cerebral cortex versus corpus callosum. We show that within the SVZ, cortical damage from mild CCI increases the number of cells fate labeled with YFP. In contrast, corpus callosum damage from TAI produces an initial decrease of YFP-labeled cells in the SVZ. These studies also demonstrate differential Shh pathway utilization between DCX neuroblasts and NG2 oligodendrocyte progenitors in the normal adult that is maintained during the response to injury.

### Neuroblast and Oligodendrocyte Progenitor Response in TBI Models

Prior studies in experimental TBI have examined the SVZ response based on proliferation and the frequency of immature cell types, such as DCX neuroblasts and NG2 oligodendrocyte progenitors. Studies with various severities of the CCI model have shown increased BrdU incorporation during the first few days postinjury (Ramaswamy et al., 2005; Radomski et al., 2013). A small proportion of these proliferating cells may be DCX neuroblasts that migrate from the SVZ or proliferate locally and are present in the lesion area of the cortex at 3 days postinjury (Ramaswamy et al., 2005; Yi et al., 2013; Susarla et al., 2014). However, these BrdU-labeled DCX neuroblasts are not maintained, which may indicate poor survival or loss of labeling due to proliferative dilution of detectable BrdU and reduction of DCX with differentiation (Yi et al., 2013; Susarla et al., 2014). The current study in *Gli1-CreER^T2^;R26-YFP* mice focuses on cells that survive beyond the acute stage. Longer time points of 2 and 6 weeks after CCI are used for this analysis of cell fate after CCI to include a sufficient postinjury interval for cell migration and differentiation. In addition, the parameters used are classified as mild CCI (Washington et al., 2012; Yi et al., 2012). DCX neuroblasts were not observed in cortical lesion areas at 2 or 6 weeks postinjury. This mild CCI may not have been sufficient to recruit DCX neuroblasts into cortical lesions. We cannot distinguish whether DCX cells may have been present at earlier time points. However, YFP heritable labeling was not observed in cortical cells with a neuronal morphology, which argues against differentiation and productive integration of Shh-responsive neuroblasts as contributing to cortical cell replacement after mild CCI.

NG2 oligodendrocyte progenitors rapidly exhibit reactive changes to even mild levels of CNS damage (Levine, 1994; Hughes et al., 2013). In the CCI model, NG2 cells have previously been shown to exhibit a strong reaction in the lesion penumbra (Chen et al., 2003) and are the most highly proliferative of the cortical cell types (Susarla et al., 2014). Because the NG2 progenitors rarely double labeled with YFP, further study of NG2 in the CCI model would have been equivalent to these prior studies and was not pursued. Instead, our analysis focused on the NG2 response in the closed head TAI model. NG2 progenitors are significantly increased in the cortex in the current TAI model, reflecting the high sensitivity of NG2 cells to changes in cortical homeostasis (Hughes et al., 2013). Our prior study of TAI characterized the glial and myelin changes in C57BL/6 mice and showed that NG2 progenitors proliferate and are increased in the corpus callosum at 1 week postinjury (Sullivan et al., 2013). In *Gli1-CreER^T2^;R26-YFP* mice, after TAI, the total NG2 progenitor population also increased in the corpus callosum but was not significantly different at 2 weeks postinjury. The attenuated NG2 response in the corpus callosum of *Gli1-CreER^T2^;R26-YFP* mice may reflect the longer time point examined here or a milder TAI severity, as the extent of axon damage in the *Gli1-CreER^T2^;R26-YFP* mice was approximately half the level observed in C57BL/6 mice when compared at 3 days postinjury.

Interestingly, SAG injection into the corpus callosum demonstrated a potent effect of Shh signaling to increase YFP cells and NG2 progenitors in the SVZ of *Gli1-CreER^T2^;R26-YFP* mice. However, SAG did not result in YFP double labeling of NG2 progenitors. It is unlikely that the increase of NG2 cells in the SVZ, and less robustly in the corpus callosum, was due to injection artifact. The density of cortical NG2 cells was not significantly different with SAG versus vehicle or in comparison between the ipsilateral and contralateral sides. Therefore, the microinjection technique did not cause a significant NG2 reaction from the pipette penetrating the cortex.

### Shh Signaling in Cortical Brain Injury

Shh signaling contributes to regenerative responses after damage to diverse regions in the CNS, including the cerebral cortex and corpus callosum, which are the target sites compared in this study. Cortical cryoinjury in *Gli1*-luciferase reporter mice showed maximal Shh pathway activation at 3 days after injury with normalization by 2 weeks (Amankulor et al., 2009). Shh in these cryolesions induced proliferation of astrocytes and Olig2-labeled oligodendrocyte lineage cells that was inhibited by cyclopamine (Amankulor et al., 2009). After cortical stab wound, Shh protein levels were highest at 3 days after injury (Sirko et al., 2013). Shh signaling produced *in vitro* conversion of cortical astrocytes to neural stem cells (Sirko et al., 2013). Shh pathway activation by SAG was sufficient to further promote *in vivo* proliferation of reactive astrocytes in the stab wound lesion (Sirko et al., 2013). Our CCI data in *Gli1-CreER^T2^* mice differ from these prior studies in that heritable labeling for Shh pathway activation was reduced in the cortical lesion area at 2 weeks postinjury, and this was maintained through 6 weeks. Shh-stimulated proliferation of astrocytes may require breakdown of the blood–brain barrier (Sirko et al., 2013). Our mild CCI is expected to cause breakdown of the blood–brain barrier but should not compromise the dura. The mild injury and intact dura may contribute to the differences from the cryolesion and stab wound studies. Alternative mechanisms include differential involvement of the Shh pathway, either the timing or the specific signaling components, that is revealed by the fate labeling in *Gli1-CreER^T2^* mice. Furthermore, the specific effect of Shh signaling may be highly dose and context dependent relative to the reactive characteristics exhibited by astrocytes, with the reduction of Smo signaling inducing reactive changes in normal adult mice (Garcia et al., 2010), while SAG administration increased proliferation of reactive astrocytes after stab wound injury (Sirko et al., 2013).

### Shh Signaling in White Matter Pathology

GLI1 expression is relatively low in the corpus callosum of noninjured adult mice (Ferent et al., 2013; Petrova et al., 2013), which is consistent with the striking lack of *Gli1* heritable labeling using *Gli1-CreER^T2^* driver mice (Garcia et al., 2010; current study). In contrast, experimental demyelination in the corpus callosum has been reported to induce broad reactivation of the Shh pathway that promotes oligodendrocyte survival as well as the proliferation and differentiation of oligodendrocyte progenitors (Ferent et al., 2013). Smo expression was reported to increase in the demyelinated lesions in microglia and oligodendrocyte lineage cells, with a subset of Olig2 cells also expressing GLI1 (Ferent et al., 2013). However, our TBI model targeting the corpus callosum induced axon damage but not *Gli1* fate labeling. Furthermore, SAG injection directly into the corpus callosum induced heritable *Gli1* labeling in the SVZ but not in the cells of the corpus callosum. These findings could indicate that SAG requires additional signaling partners to be effective within corpus callosum cells, akin to the requirement hypothesized for blood–brain barrier disruption for the Shh conversion of cortical astrocytes into neural stem cells (Sirko et al., 2013). Another possibility is that *Gli1* transcriptional activation may not be the predominant response to Shh signaling among the oligodendrocyte lineage cells and other cell types within the corpus callosum. Indeed, the level of *Gli1* transcriptional activation may reflect an early stage of neuronal commitment with GLI1 versus oligodendrocyte lineage commitment without GLI1. Consistent with this possibility, NG2 cells were rarely double labeled with YFP. Furthermore, YFP cells were transiently reduced in the SVZ after TAI, which may divert slow cycling or transit amplifying populations of neural stem cells toward the oligodendrocyte lineage. An additional consideration is that SAG injected into the corpus callosum may act by signaling through other cell types that secrete factors to increase NG2 progenitors. In this way, the Shh pathway could indirectly modify the NG2 progenitor response in lesioned tissues.

Canonical Shh signaling is dependent on the balance of activator and repressor effects among the *Gli* family members (Briscoe and Therond, 2013). *Gli1* expression serves as a readout for activation of the Shh signaling pathway. GLI2 acts as a transcriptional activator of *Gli1* in the presence of high levels of Shh. GLI2 is expressed in normal adult mouse corpus callosum (Ming et al., 2013; Petrova et al., 2013), while GLI3 is not detected (Ferent et al., 2013; Ming et al., 2013). Whether GLI2 levels modulate the oligodendrocyte lineage response to corpus callosum damage is not clear. Two studies of experimental demyelination with lysolecithin injection into the corpus callosum do not corroborate one another readily. GLI2 mRNA transcripts were increased in lesion areas after 10 days (Ferent et al., 2013). However, GLI2 protein was decreased at 3 days’ postlesion induction (Ming et al., 2013). In spinal cord, GLI1 expression was observed in NG2 cells of normal adult mice but downregulated in demyelinated lesions (Wang et al., 2008). Further studies will be important to delineate the potential role of GLI2 and modulation of GLI1 in the oligodendrocyte lineage in the normal adult CNS and in response to white matter damage. Based on the current findings and these prior studies, Shh signaling in the oligodendrocyte lineage may occur via modulation of repressive effects of GLI2, given the lack of *Gli1* transcriptional activation observed in *Gli1-CreER^T2^;R26-YFP* mice. However, more complex interactions may regulate the response of oligodendrocyte lineage cells to Shh. Smo is expressed at 4.7 fold higher levels in oligodendrocyte progenitors compared with myelinating oligodendrocytes (Cahoy et al., 2008). Conversely, the expression of hedgehog interacting protein increases 17.2 fold with oligodendrocyte progenitor differentiation into myelinating oligodendrocytes (Cahoy et al., 2008). Interestingly, hedgehog interacting protein sequesters hedgehog ligand and limits diffusion (Briscoe and Therond, 2013), which could limit Shh signaling in the white matter as observed in our data.

### *Gli1* Transcriptional Activation in the SVZ

In the SVZ of noninjured adult mice, *Gli1* transcriptional activation is Smo dependent and strongest in ventral neural stem cells that then migrate into the dorsal SVZ (Ihrie et al., 2011; Petrova et al., 2013). GLI2 is expressed throughout the dorsal–ventral extent of the SVZ (Petrova et al., 2013). However, GLI3 appears to have a predominant role in modulating the Shh pathway in the SVZ neural stem cells. In the absence of Shh, GLI3 acts as a repressor. Shh is required for the negative regulation of this repressive GLI3 effect to allow for normal proliferation of the slow cycling neural stem cells in the adult SVZ (Petrova et al., 2013). The heritable labeling in our TBI studies using *Gli1-CreER^T2^* driver mice was designed to fate label based on Shh pathway activation with tamoxifen on Days 2 and 3 to capture the early response to injury. The mice survived through at least 2 weeks postinjury to allow time for migration and differentiation. Different approaches would be required to identify the specific cell stage at the time of fate labeling, as the extent of recombination among different cell populations was not detected accurately at shorter time points using two different reporter constructs (Garcia et al., 2010; data not shown).

## Conclusion

Optimizing the repair capacity of the adult CNS will require modulating the complex interplay among *Gli* family members that impact the regenerative potential from the SVZ with consideration of the differential response between neuronal and oligodendroglial lineages. Further studies will be important to address the therapeutic potential of short-term Shh pathway agonist administration, which may also act through enhancing cell replacement in the cortex (Sirko et al., 2013), inducing oligodendrocyte differentiation in white matter (Ferent et al., 2013), promoting blood–brain barrier integrity, and modulating the immune response (Alvarez et al., 2011). The data presented highlight the need to more fully appreciate the specificity of the neural stem cell and progenitor response to distinct forms of cell and tissue damage. This analysis is particularly relevant to TBI in which the patients may have extremely different involvement of neuronal cell bodies in the cerebral cortex relative to traumatic axonal injury in white matter tracts.

## Summary

Sonic hedgehog-responsive cells were fate mapped after traumatic brain injury in *Gli1-CreER^T2^;R26-YFP* mice. *Gli1* labeling in the subventricular zone increased after cortical injury but decreased with corpus callosum damage. *Gli1* labeling was prevalent in neuroblasts but extremely rare in oligodendrocyte progenitors.
